# Osteosarcoma: a review of current and future therapeutic approaches

**DOI:** 10.1186/s12938-021-00860-0

**Published:** 2021-03-02

**Authors:** Xin Zhao, Qirui Wu, Xiuqing Gong, Jinfeng Liu, Yujie Ma

**Affiliations:** 1 Anhui Chest Hospital, 397 Jixi Road, Hefei, 230022 China; 2 Materials Genome Institute, Shanghai University, Shanghai, 201800 China

**Keywords:** Osteosarcoma, Staging, Diagnosis, Treatment

## Abstract

Osteosarcoma (OS) is the most common primary bone malignancy that affects children and young adults. OS is characterized by a high degree of malignancy, strong invasiveness, rapid disease progression, and extremely high mortality rate; it is considered as a serious threat to the human health globally. The incidence of OS is common in the metaphysis of long tubular bones, but rare in the spine, pelvis, and sacrum areas; moreover, majority of the OS patients present with only a single lesion. OS has a bimodal distribution pattern, that is, its incidence peaks in the second decade of life and in late adulthood. We examine historical and current literature to present a succinct review of OS. In this review, we have discussed the types, clinical diagnosis, and modern and future treatment methods of OS. The purpose of this article is to inspire new ideas to develop more effective therapeutic options.

## Background

Osteosarcoma (OS) is a malignant tumor that originates in the mesenchymal tissue (which constitute spindle-shaped stromal cells that can produce bone-like tissues), and it accounts for 20% of all cases of primary malignant bone tumors in the world [1, 2]. In fact, it is the most common type of primary malignant bone tumor among adolescent patients [3–5]. The incidence of OS is common in the metaphysis of long tubular bones (such as the proximal humerus, the distal femur, and the proximal tibia), but rare in the spine, pelvis, and sacrum areas [6]. The majority of patients with OS present with only a single lesion [7]. Clinically, the onset of the disease is characterized mainly by local pain and swelling, and occasionally by joint dysfunction. A few patients have also been treated for pathological fractures. The symptoms of growth pain and trauma are confounding, but the degree of malignancy is high [8]. Notably, nearly 10–20% of the patients are affected by measurable metastatic disease before actual onset, the most common site being the lungs (85%), followed by the bones (8–10%) and, occasionally, the lymph nodes. The remaining 80–90% of the patients can be considered to possess subclinical or micrometastases, which cannot be detected accurately by using the presently available diagnostic methods [9]. The presence of metastatic disease is a clear indication of poor prognosis of OS [10]. In fact, the prognosis of such patients depends almost entirely on the metastasis and drug resistance, especially the status of lung metastases [11]. In addition, it is estimated that 60% of the OS patients do not show significant lung metastases at the time of initial examination, although micrometastases exists [12]. It is therefore very critical to perform detection in timely and early manner using highly sensitive diagnostic approaches of micrometastasis toward improving the overall survival of OS patients. Notably, the incidence of OS has a bimodal distribution pattern with respect to age: it first peaks during the age of 10–14 years (the adolescent development phase) and then a second time after the age of 60 years [13, 14].

The causative factor in most cases of OS remains unclear. Cytogenetic studies suggest that, in OS, complex changes occur in some chromosomes, although no specific pattern has yet been recorded. The involvement of two genes, one with a genetic mutation associated with retinoblastoma at 13q14 and the other with autosomal recessive p53 mutation associated with Li-Fraumeni syndrome at 17p13, are currently attributed to the progressive accumulation of genetic defects leading to OS [15, 16].

In the 1970s, the standard treatment of OS was amputation, although the 5-year survival rate post-amputation was < 20% [17]. In the 1980s, the advancement in surgical techniques, research on effective chemotherapy drugs, and preoperative and postoperative chemoradiation adjuvant treatment, among other significant developments helped improve the OS treatment modality. For instance, limb salvation gradually replaced the conventional approach of amputation. Notably, the combined limb salvage treatment supported with neoadjuvant chemotherapy achieved great clinical success by enabling patients to survive at least 5 years without subjecting them to the sufferings of amputation. The survival rate also increased to nearly 80% [18]. In the 1990s, gene therapy became a research hotspot and provided new insights to the treatment of OS. In the new century, with the successful large-scale sequencing of the human genome, stem cell research gradually became the most dynamic, influential, and promising field in life science research. Several studies confirm that stem cells play an important role in the mechanisms of tumorigenesis, development, metastasis, and relapse, including those in OS [19]. The present article is a literature review of the types, staging, clinical diagnosis, and modern treatment methods of OS. New methods for treating this disease have also been discussed. And we hope this review will provide readers a general understanding of current status in OS, and inspire further investigations in OS treatment.

## Type and staging

OS is classified based on its location, involved cell type, and tumor grade. Most OS are located at the center of the long bone marrow cavity [5, 20]. OS can also affect the periosteum, cortex, soft tissue, or preexisting bone lesions. All OS contain varying amounts of osteoid, most of which are constituted of cartilage and fibrous tissues [21, 22]. Depending on its type, if a particular cell type constitutes 50% of a malignant tumor, that tumor is considered to be an osteoblast, chondroblast, or fibroblast, accordingly. The prevalence rates of these three cell types are 50–80%, 5–25%, and 7–25% [21, 23, 24]. In addition, the histological grades are low (grade 1), medium (grade 2), and high (grade 3 or 4), which are distinguished based on the tumor area with the highest degree of degeneration and the highest mitotic rate [21, 23]. Table 1 [21] lists the various types of OS. Moreover, it has been reported that 30% of chondroblasts or osteoblasts and well-differentiated OS usually respond poorly to chemotherapy [25]. In addition, some studies have reported that the metastatic-free survival rate of tumors with chondrocyte subtypes is reduced [26]. It has also been reported that the survival rate of high-grade OS is independent of its dominant cell type [21].

**Table 1:** Types of OS

1. Central OS	2. Multifocal
a. Conventional	3. Gnathic
i. Osteoblastic	4. Surface OS
ii. Chondroblastic	a. Periosteal
iii. Fibroblastic	b. Parosteal
b. Epithelioid	c. High-grade surface
c. Giant cell-rich	d. Intracortical
d. Osteoblastoma-like	5. Secondary OS
e. Small cell	
f. Telangiectatic	
g. Low-grade central	

Cancer staging helps determine the extent or potential of a tumor to spread-out in the body. It also provides a means for predicting the possible prognosis. As described by Enneking, a good staging system should make it easier for doctors to communicate a patient's condition, suggest prognosis, guide surgical treatment, and suggest appropriate adjuvant treatment [27]. Currently, the two most commonly used surgical staging systems for OS and malignant OS are the Enneking/MSTS (Table 2 [27]) and AJCC (Table 3 [28]) systems. Despite the presence of some subtle differences between these two systems, most of the basic concepts are the same as they depend on the grade, size, and metastasis of the tumor [29].

**Table 2:** Enneking/MSTS staging system

Stage	Grade	Size	Metastasis
IA	Low	*T*1—intracompartmental	M0—none
IB	Low	*T*2—extracompartmental	M0—none
IIA	High	*T*1—intracompartmental	M0—none
IIB	High	*T*2—extracompartmental	M0—none
III	Any	Any	M1—regional or distant

**Table 3:** AJCC staging system for bone sarcoma

Stage	Grade (G)	Size (*T*)	Lymph node (N)	Metastasis (M)
IA	G1—low	*T*1 < 8 cm	N0—none	M0—none
IB	G1—low	*T*2 > 8 cm	N0—none	M0—none
IIA	G2—high	*T*1 < 8 cm	N0—none	M0—none
IIB	G2—high	*T*2 > 8 cm	N0—none	M0—none
III	Any G	Any *T*	Skip metastasis	Skip metastasis
IVA	Any G	Any *T*	N0—none	M1—lung metastasis
IVB	Any G	Any *T*	N1—lymph node metastasis or N0	M1—non-lung metastasis

### Imaging and diagnosis

*X-rays* [30], with high spatial resolution, can comprehensively and intuitively display the size and location of the tumor as well as the extent of bone destruction caused; it also helps visualize even slight periosteal reactions and the Codman triangles [31]. The radiation dose received by a patient during X-ray examination is small, and causes only minimal damage to the body in a single examination. The technical operation of X-ray is simple and user-friendly. In addition, as it is relatively affordable by most patients, it is the preferred tool for preliminary screening of lesions and for diagnosis [32]. Although X-ray examination offers several advantages, it also involves some shortcomings. For instance, it has a low-density resolution. It does not clearly display tiny bone damages and soft tissue masses as well as the tumor invasion of bone marrow and callus, soft tissue masses, and the surrounding structures [33]. These pitfalls in its use for primary screening have affected the early diagnosis and staging of tumors.

*Computed tomography (CT) *[34] is used to visualize the extent of invasion inside and outside as well as to detect micromineralized bone-like formation of tumors, which is not visible on X-rays [35]. CT is also useful in the diagnosis of pathological fractures. CT is the best imaging method to visualize complex bones, vertebrae, and craniofacial bones, which often occur in the elderly. Owing to its multi-planar and three-dimensional imaging capabilities, CT also helps with preoperative planning [36]. As patients with OS often develop lung metastases at an early stage, it is recommended that chest CT examination be performed at the early stage to confirm the presence or absence of lung metastatic lesions. CT offers the sensitivity of 75% and the specificity of nearly 100% to detect lung metastases [37]. A chest CT scan is considered as the reliable imaging tool, albeit its two limitations: (i) not all lung nodules detected during surgery can be determined by CT and (ii) not all nodules detected on CT are true metastatic lesions (especially when the lesion size is < 5 mm) [38].

*Magnetic resonance imaging (MRI)* [39] is an important tool to determine the cancer stage and hence in preoperative planning for the surgical treatment of OS, because it can accurately display the intramedullary range of tumors, the size of soft tissue masses, and the surrounding structures [35, 40]. These observations enable a surgeon determine the appropriate edge to plan bone resection [2]. MRI also demonstrates the involvement of tumors in the joints or in invasion to the neurovascular structures, helps accurately define the surgical boundary, and provides a reference for the feasibility of limb salvage surgery [38, 41]. MRI of the entire affected bone is important as it helps assess the presence or absence of skip metastases. Jump metastasis is a small, non-adjacent lesion that is usually located in the proximal intramedullary canal of the affected bone. Although larger lesions can be easily visualized on X-rays, MRI is the most sensitive approach for detecting their presence [42]. The detection of a jump transfer is critical for two main reasons: (i) when a part of a bone is to be included in the surgical resection and (ii) when the lesion is located in the same bone; the latter case is considered as a site of distant metastasis in terms of staging and has a negative impact on the prognosis [43]. Table 4 [44] introduces the comparison of imaging findings. The number of ‘+’ represents the degree of accuracy in the diagnosis of a feature.

**Table 4:** Comparison of imaging findings

Imaging	Bone destruction	Periosteal reactions	Size of range	Soft tissue masses	Tumor bone	Codman triangles
X-rays	++	++++	++	++	++	++++
CT	++++	+	++++	+++	++++	++
MRI	++++	++	++++	++++	++++	++

For patients with suspicious OS, a cost-effective X-rays examination should be performed first, followed by CT or MRI of the lesion to further evaluate the extent of tumor involvement. CT can find details, and has obvious advantages in showing osteosarcoma lesions with complex structures and many overlapping parts, supplementing signs that cannot be shown by x-rays. However, CT may have missed diagnosis of skip metastases of osteosarcoma. The extent and stage of tumor invasion of bone marrow is not as good as MRI, and MRI can accurately show the positional relationship between tumor and adjacent soft tissues, joints and vascular bundles. However, the specificity of MRI signal is not high, and the display of periosteal reaction and Codman triangles is not as good as CT. Therefore, the combination of their respective advantages can effectively guide the choice of clinical procedures to improve the treatment effect of patients and ensure a good prognosis.

In addition, a few more imaging technologies have been developed recently, including positron emission technology (PET) [45] scanning and dynamic-enhanced MRI (DCE-MRI) [46]. Although these technologies are not included in the current routine imaging protocols for OS, researchers are making efforts to determine their possible role in the future diagnosis, monitoring of treatment response, and in detecting relapse [29].

*Biopsy* is essential in the accurate diagnosis of OS [40, 47]. In fact, the current gold standard for the diagnosis of OS remains tissue biopsy [2]. However, the currently available imaging findings cannot support the diagnosis of benign results, which makes histopathological examination particularly important at the present time. Pathological research can yield a deeper understanding about the occurrence, development, and outcomes of tumors. Performing tissue biopsy offers the following advantages [2, 48]: (i) enables intuitive analysis and observation of lesion tissues; (ii) allows more accurate understanding of the trends and development of lesions and the processes involved; and (iii) helps understand the body's ability to resist disease. The common biopsy methods include needle aspiration biopsy and incisional biopsy. The former is relatively easy to perform, but occasionally results in unfavorable puncture and material acquisitions; also, tumor tissues are not effectively obtained for diagnosis by this approach [49]. Hemostasis is the most important aspect of biopsy operation. Puncture biopsy is usually performed at the puncture point for 2–10 min to stop the bleeding, while electrocoagulation hemostasis is often employed in open biopsy procedures [50]. Therefore, it is extremely important to select an appropriate biopsy method so as to avoid delays in timely treatment of patients as a result of missed or overlooked diagnosis, which may lead to the loss of opportunity for surgery or may affect the prognosis [51].

## Treatment

### Surgery

Tumor surgery to extensively remove the tumor is conducted with the aim of achieving complete resection of the disease. In this case, the surgery can be of two types: limb salvage and amputation [52]. Amputation is an important treatment approach for early OS cases. Following ineffective adjuvant therapy, amputation is deemed as a necessary and effective treatment alternative for malignant bone tumors that can cause extensive cell destruction [53]. For amputation, the osteotomy plane requires a tumor-free border of at least 5 cm [54]. Most physicians believe that the osteotomy safety plane is 5-cm outside of the tumor plane. However, there is a need for a reliable reference standard to the dimensions for determinations based on X-rays, CT, and MRI. The aim of surgical treatment for OS has evolved from saving lives to maximizing the functions of the affected limbs [55]. Limb salvage surgery refers to the surgical procedure to restore bone and joint function after extensive resection of malignant bone tumors of the limbs [56]. The key to the operation is to select the appropriate boundary [57]. With the recent popularization of comprehensive limb salvage therapy in combination with neoadjuvant chemotherapy, limb salvage surgery has been used more often in clinical applications. In fact, 80–95% of all patients with soft tissue sarcoma of the bones and limbs can undergo limb salvage surgery. Although the incidence rate of local recurrences of amputations and limb salvage are the same, limb salvage patients have a higher 5-year survival rate [58]. Limb salvage surgery preserves the patient’s apparent integrity not only functionally, but also externally [59]. OS surgery should completely remove the lesion to avoid local recurrence and distant metastasis. If the lesion is not completely removed during the operation, the local recurrence rate can be as high as 25% [60]. In recent years, ablation has been gradually applied to limb salvage surgery for OS and has achieved good clinical effects [61]. Tumor ablation refers to the use of physical or chemical methods to remove tumor cells. There are two methods of ablation: temperature ablation and chemical ablation [62]. After a tumor is removed, there is a possibility of larger bone and soft tissue defects being formed. With the advancement in bone tissue engineering and in materials science, it is now possible to reconstruct the bone and soft tissue defects formed after tumor resection through tumor bone inactivation replantation, allogeneic bone transplantation, autologous bone transplantation, and artificial prosthetic replacement [63]. Each reconstructive method has advantages and disadvantages after a tumor resection. Through the artificial prosthesis replacement the limb function can be quickly restored; however, there is a risk for long-term device loosening and wear. A key advantage of many allografts is that tendons and ligaments remain attached to the graft bone for host soft tissue attachment. A key advantage of many allogeneic bone transplantations is that the tendons and ligaments are still attached to the graft bone for host soft tissue attachment. The disadvantages of allogeneic bone transplantation are the risk of fracture, bone nonunion, joint instability, and osteoarthritis of joint reconstruction. Autologous bone transplantation share the nonjoint-related concerns. The advantages of tumor bone inactivation replantation are simple operation, no need to consider the problem of bone matching, and save bone replacement materials. Its disadvantage is that pathological fractures are prone to occur in the process of bone tissue repair and reconstruction [64]. Computer-assisted tumor surgery is becoming increasingly important in the management of OS. Currently, there is no commercially available platform that meets all of the software and hardware needs relevant to surgery for OS [65]. This will be the direction of future research.

### Chemotherapy

Studies on the chemotherapeutic effect of OS had begun in the 1970s. At that time, chemotherapy was used as an adjuvant treatment after surgery to eliminate the formation of lesions and metastases that could not be completely removed by surgery alone [66]. In the late 1970s, in order to eliminate the subclinical nature of tumors before surgery, to reduce the surrounding reaction zone, and to create a suitable condition for limb salvage surgery, innovative preoperative chemotherapy was boldly and successfully applied in the clinic; this approach came to be known as neoadjuvant chemotherapy [67]. The significance of neoadjuvant chemotherapy is that it allows early systemic treatment to eliminate potential micrometastases; allows evaluation of preoperative chemotherapy based on tumor necrosis rate to guide postoperative chemotherapy; reduces tumor edema bands; increases the limb salvage rates; and reduces the recurrence rates [68]. This concept was widely accepted and then widely used in clinical practice, gradually forming a comprehensive limb salvage treatment complimenting neoadjuvant chemotherapy, making limb salvage surgery the mainstream for OS and significantly improving the 5-year survival rate of OS [69]. The concept of neoadjuvant chemotherapy has become a milestone in the history of OS treatment, and this concept continues to be used. Chemotherapy drugs for OS have been updated since the 1970s. As such, presently, the most commonly used chemotherapeutic drugs with known high efficacy are adriamycin (ADM), high-dose methotrexate (HDMTX), cisplatin (DDP), and ifosfamide (IFO) [8, 66]. Multiple chemotherapy regimens can be combined by pairing the above chemotherapy drugs according to different doses and usage sequence. Adjuvant MAP chemotherapy remains a cornerstone of therapy, it is composed of HDMTX, ADM, DDP combined application [70]. Most hospitals worldwide conduct 2–6 courses of preoperative chemotherapy for a total of 6–18 weeks [71]. The toxic and side effects of chemotherapeutics can also not be ignored, which includes incidents such as liver and kidney function damages, bone marrow suppression, neurotoxicity, and gastrointestinal reactions, among others [72]. Unfortunately, it has been reported that ADM-induced cardiomyopathy may be permanent [73]. Moreover, cisplatin may cause high-frequency hearing loss in up to 11% patients [74]. Currently, manifold research and demonstration are active for basic experiments and clinical trials, with the superposition of drug types, doses, and toxic side effects being the main focus. However, the purpose of these researches is to improve the efficacy and the survival rate and to reduce the damage caused by the side effects of treatment methods to the human body [75, 76]. Vascular interventional therapy adopts arterial infusion of chemotherapy drugs and microsphere embolization to treat osteosarcoma, which has the characteristics of small systemic dose and large local dose. The limitation of anticancer drugs has been improved by the use of nanocarriers [77]. Various nano-platforms capable of delivering the chemotherapy drugs rightly to the tumor site have been developed to improve the therapeutic effects and minimize side effects, but most of them are still at experimental stage. This will become the future development direction of chemotherapy in clinical application [78].

### Radiotherapy

For patients who cannot be surgically resected or in whom tumors remain on the resection margin, as well as for patients with OS in whom the tumors respond poorly to chemotherapy, local radiotherapy has been found to create a certain impact [79, 80]. Early results confirmed that external irradiation along with systemic therapy may act as a successful approach toward local control and symptom relief [81]. After using induction chemotherapy effectively for non-metastatic OS of the limbs, Machak et al. [82] believes that radiotherapy is a reliable method to control local diseases and protect limb functions. Ciernik et al. [83] demonstrated that proton therapy provides a high-dose radiation therapy for local treatment of patients with unresectable or incompletely removed OS. However, OS is not sensitive to radiotherapy. In fact, radiosensitizers have become a new hotspot in clinical research in the recent times. Radiosensitizers can increase the sensitivity of tumor cells to radiotherapy without harming the normal tissues as well as promote radiation to kill tumor cells with high safety [84]. Recent studies have confirmed that the combined use of ginseng polysaccharide (GPS) and ionizing radiation (IR) can increase the sensitivity of OS cells to IR [85]. Although sensitizers can serve as a new breakthrough point in radiotherapy, the advancement and improvement of radiotherapy technology and equipment has helped increase the number of long-term surviving tumor patients [86]. In the future, radiotherapy for OS will be based on radiotherapy sensitization research, combined with advanced techniques such as stereotactic radiotherapy [87], proton radiotherapy [88] and heavy ion radiotherapy [89] and organic combination of surgical treatment and chemotherapy, to achieve better treatment effect at a low dose and high precision. It role in comprehensive limb salvage adjuvant treatment cannot be ignored.

### Immunotherapy

**Fig. 1:**
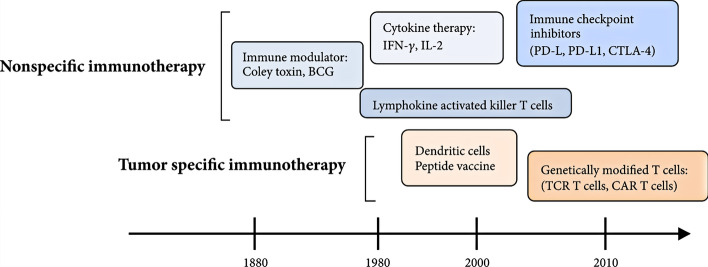
Development of immunotherapy for malignancies

Immunotherapy is performed to regulate the immune function of a body to enable killing of tumor cells, regulation and balancing of the body's immune function, and differentiating and inhibiting tumor growth, among others [90]. This treatment approach has gained importance in the adjuvant treatment of tumors owing to its specific and effective outcomes for cancer patients, especially by providing a new and effective treatment method for advanced, metastatic, and recurrent OS [91, 92]. As the most basic elements in immunotherapy, cytokines regulate the activation, proliferation, and functional activity of immune cells [93]. Immunotherapy of OS includes non-specific immunotherapy, specific immunotherapy, adoptive immunotherapy, and immuno-guided therapy [94]. Tumor immune responses have been reported for over 100 years (Fig. 1) [95]. Presently, interleukin-2 has been used for postoperative treatment of OS to yield clinical effects. Interleukin 2 activates effector T cells and enhances the function of natural killer cells [96]. Moreover, T cell-mediated cellular immunity majorly contributes to the body's anti-tumor immune effect, while natural killer cell-mediated natural immunity acts as the body's first-line of defense against tumors [97]. Checkpoint inhibitors are also an attractive research area [98]. However, with the improved understanding of tumor immunity, it is now confirmed that tumor cells have low immunogenicity and hence cannot express strongly to the body's immune system. Therefore, if immunogen-related molecules are introduced and then expressed into tumor cells, the immunogenicity of tumor cells can be enhanced, resulting in the production of a strong immune stimulation to the body's immune system; this line of thought has given rise to a new subject in tumor immunotherapy [95]. Although there is still a lot of work to be done, it is hoped that immunotherapy can bring breakthroughs and revolutionize the treatment of OS.

### Gene therapy

Considering that the most fundamental cause of OS is genetic mutation, the involvement of genetic research is crucial in its prevention and cure strategies [99]. Gene therapy is a biomedical method that introduces normal genes or genes with therapeutic effects into human target cells through vectors to correct the gene defects or exert therapeutic effects sufficient to achieve therapeutic outcomes [100]. OS gene therapy is mainly focused on tumor suppressor genes, suicide genes, combined gene therapy, antisense genes, immune genes, and anti-angiogenic genes [101]. Presently, tumor suppressor genes p53, p16, p21, and Rb have been tested for the treatment [102]. Among these, *p53* has been studied in-depth. These studies suggest that patients with OS often have mutations in their *p53* [103]. Wu et al. [104] noted that p53-expressing protein may become a prognostic biomarker for predicting the overall survival of OS, which would further deepen the status of p53 as an entry point for gene therapy of OS. Ye et al. [105] report that the overexpression of wild-type p53 increases the sensitivity of chemotherapy to multidrug-resistant OS cell lines, which in turn can provide new clues to resolve chemotherapy resistance. The thymidine kinase (TK)/propoxyguanosine (GCV) system is preferred for the treatment of suicide genes. Zhang et al. [106] first transfected lipid-mediated *TK* into the OS cell line MG-63 and then added GCV; this approach could successfully inhibit the growth of the OS cell line MG-63. Interestingly, the increase of GCV concentration led to an increase in the rate of apoptosis. This finding confirms the broad clinical application prospects of the TK/GCV suicide gene system. Leinonen et al. [107] conducted supplementary experiments on this system. They found that *TK* transfection alone could not inhibit tumor growth and that after 1 week or more of GCV addition could induce the “bystander effect” [108], resulting in significantly killing the tumor cells. The effect of combined gene therapy was found to be better than other gene therapies on OS. On the basis of combined gene therapy, the effect of combining other treatment methods has been found to be more significant, not only by producing synergistic effects but also by reducing the adverse reactions caused by the use of a single drug. The combination of gene therapy with other treatment methods for treating OS patients is expected to gain recognition as a meaningful approach to gene therapy in the future [109], especially genetically modified T cell therapy. It has shown promise in preclinical studies.

In the recent years, despite that gene therapy has made great progress and has delivered valuable prospects, it is still in its experimental stage, and hence a long way from actual clinical application.

## Conclusion

OS is a malignant tumor that is derived from the mesenchymal tissues. Advances in chemotherapy and surgery have made it possible to transform OS from an almost universally fatal disease to a disease that most patients can survive. Accurate and effective diagnosis, preoperative chemotherapy, surgical resection, postoperative chemotherapy, and life-long monitoring are critical factors involved in successful management of this complex and potentially fatal disease. Presently, the comprehensive treatment of OS patients is based on preoperative and postoperative chemotherapy and surgical treatment. Although the treatment outcome has improved as compared with that in the past, in recent years, the treatment of OS has encountered bottlenecks, especially for patients with lung metastasis and chemotherapy resistance. The treatment of these patients with medication requires development of new and effective drugs and innovative treatment strategies. With the continuously advancing research in the field of molecular biology, research on tumor genes has also gained momentum. Immunotherapy and gene therapy are expected to provide more opportunities and possibilities for the treatment of OS. The optimal combination and strategy of different treatment methods are also hotspots in the current research scenario. We believe that OS can be overcome in the near future with suitable research aptitude.

## Data Availability

Not applicable.

## Abbreviations

OS: Osteosarcoma
CT: Computed tomography
MRI: Magnetic resonance imaging
PET: Positron emission technology
DCE-MRI: Dynamic-enhanced MRI
ADM: Adriamycin
HDMTX: High-dose methotrexate
DDP: Cisplatin
IFO: Ifosfamide
GPS: Ginseng polysaccharide
IR: Ionizing radiation
